# Editorial: Current understanding of genomic and chromosomal instabilities in solid malignancies

**DOI:** 10.3389/fonc.2023.1245087

**Published:** 2023-08-24

**Authors:** Pavel Vodicka, Michal Kroupa, Ludmila Vodickova, Rajiv Kumar

**Affiliations:** ^1^ Institute of Experimental Medicine, Czech Academy of Sciences, Prague, Czechia; ^2^ Institute of Biology and Medical Genetics, First Faculty of Medicine, Charles University, Prague, Czechia; ^3^ Biomedical Center, Faculty of Medicine in Pilsen, Charles University, Pilsen, Czechia; ^4^ Institute of Medical Biometry and Informatics, University of Heidelberg, Heidelberg, Germany

**Keywords:** DNA damage response, mitotic regulation, telomere homeostasis, solid malignancies, genomic instability

A fundamental precondition of life is the highly precise transfer of genetic information and the preservation of genomic integrity. Hanahan and Weinberg, in their seminal review, had defined the hallmarks of cancer as acquired functional alterations that enable cancer cells to survive, proliferate, and disseminate with the development of genomic instability in cancer cells as a significant prerequisite for malignant transformation. Genomic instability, as defined, includes genetic changes that include mutations, chromosomal rearrangements, and telomerase function (1). The genomic instability in transforming epithelial cells into malignant cells remains paramount in tumorigenesis, as augmented by the accumulated data (2).

In general, multiple pathways are implicated in the maintenance of genomic integrity and in the prevention of its instability. Those pathways (**Figure 1**) predominantly include (I) DNA damage response and DNA repair mechanisms, (II) DNA replication and mitosis, and (III) telomere maintenance. The accumulation of various DNA damage due to the altered DNA repair capacity and telomere shortening disrupt the genomic integrity and pave the way to malignant transformation. DNA damage response (DDR), a signaling network that processes DNA damage, is critical for cancer progression and chemotherapy outcomes. DDR, a complex process, that detects DNA lesions and activate signaling pathways, such as cell cycle checkpoint induction, DNA repair, and or induction of cell death (3–5).

**Figure 1:**
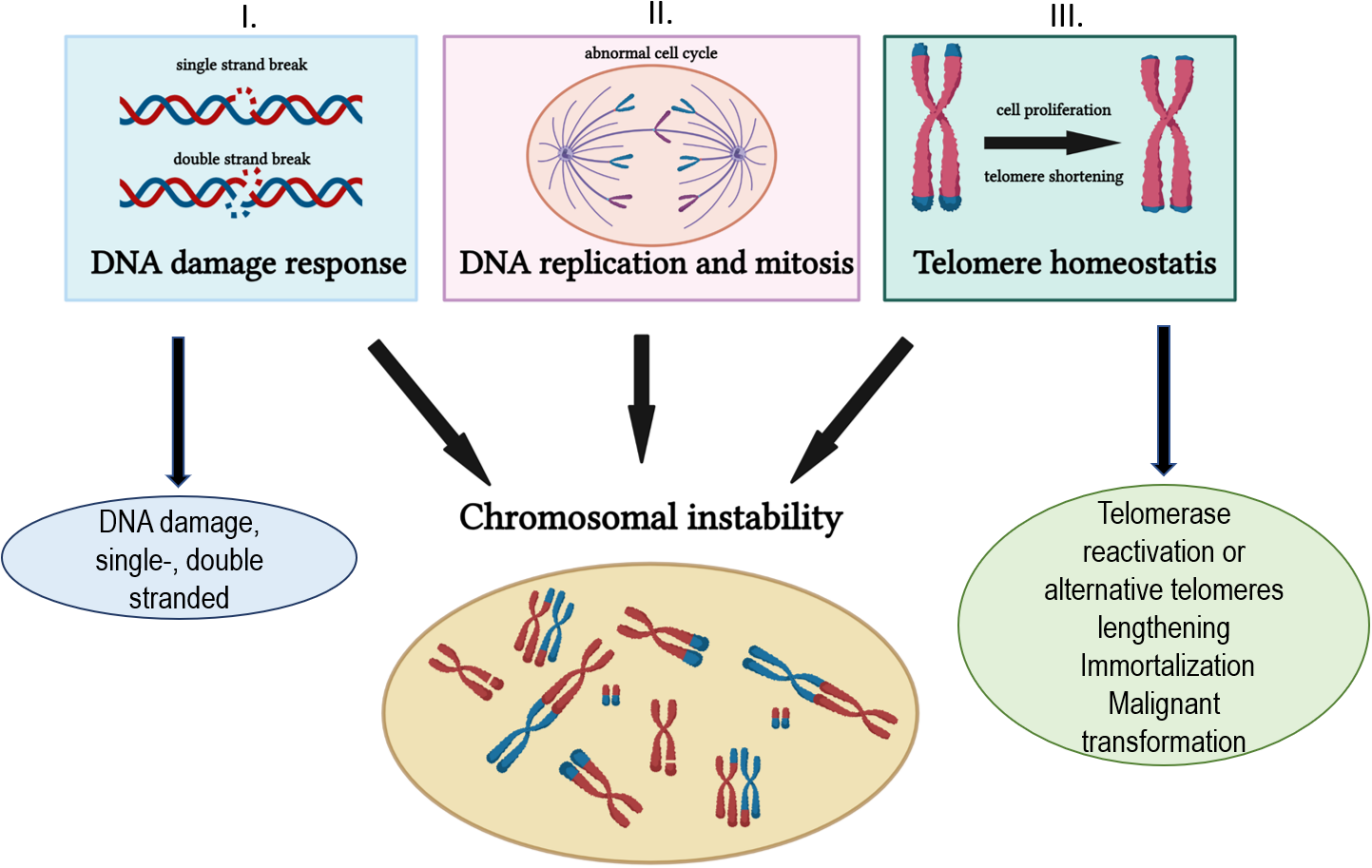
The processes involved in genomic instability.

In solid cancers, impaired DNA repair, deficiencies in mitotic regulation, and altered telomere length (TL) homeostasis are vital aberrations in cancer initiation, progression, and dissemination. As is generally believed, suboptimal DNA repair results in a broad range of critical mutations, leading to genomic instability. The functional impairment of most DDR processes is widely pervasive in different cancers (6). The familial cancers exhibit high penetrance germline mutations in DNA repair genes: BRCA1/BRCA2 in breast cancer, MMR and polymerase deficiency (MLH1, MSH2, MSH6, PMS2, and POLE genes) in colorectal and ovarian cancers (OC), deleterious mutations in RAD51C and RAD51D and BRCA1 mutation in OC exemplify that paradigm (reviewed by 6). Chromosomal instability (CIN) in cancers often occurs due to improper regulations of mitotic fidelity and faithful chromosome segregation. These processes impact mitosis and its alteration results in dysfunctional centrosomes that trigger and maintain CIN (7). Functional aberrations in the mitotic checkpoint, comprising mutations and changes in gene expression, result in abnormal chromosome content and/or aneuploidy, important players in cancer development. An improper checkpoint response is also involved in developing drug resistance to microtubule poisons employed in treating various solid malignancies for a long time (8).

Telomeres maintain genomic integrity by protecting chromosomal ends (9). However, due to the intrinsic inability to completely replicate lagging DNA strands, telomeres become progressively shortened during successive cell division. Endogenous and exogenous DNA-damaging genotoxicants, including anticancer drugs, also affect the telomere length. Because of increased proliferation, telomeric attrition is considerably faster in tumor cells than in non-malignant somatic cells. Telomere shortening doubles as a robust tumor-suppressing mechanism, limiting the lifespan of cells to prevent uncontrolled growth. On the contrary, cancer cells often develop a mechanism to surpass telomere attrition through telomerase rejuvenation that stabilizes telomeres for continued proliferation (10). The rejuvenated telomerase preferentially stabilizes the shortest telomeres and critically short telomeres, which can lead to the formation of anaphase bridges through breakage–fusion–bridge cycles that contribute to CIN (11). Therefore, ubiquitous in advanced solid cancers, telomerase is fundamental to cell immortalization.

Human solid neoplasms often exhibit structural and numerical chromosomal instability (CIN) (4, 12, 13). CIN creates abnormal aneuploid karyotypes or continually expands phenotypic heterogeneity because of the consecutive cell divisions of tumor cell populations. For instance, CIN studied in colorectal carcinoma disclosed losses predominantly on chromosomes 1p, 5q, 8p, 17p, 18p, 18q, 20p, and 22q, whereas gains mainly were identified on chromosomes 1q, 8q, 12q, 13q, and 20q. These regions are likely to harbor oncogenes or tumor suppressor genes. Several signaling pathways and genes are associated with CIN, including APC, Wnt/β-catenin, p53, TGF-β/SMAD, KRAS, BRAF, and PIK3CA. These pathways are closely linked with the progression and metastasis of colorectal carcinoma (14).

The present Research Topic includes a collection of eight articles based on experimental data and a literature survey of genomic instability in various solid malignancies. Since genomic instability is a complex and heterogeneous process, the authors have logically addressed various aspects, including genetic characteristics, new genetic markers in the process, the role of the p53 family, the complex molecular profile of DNA repair during therapy, the prognostic significance of microsatellite instability and loss of heterozygosity. Furthermore, one study analyzed clinical perspectives of microsatellite instability in metastatic colorectal cancer, and one manuscript reports the process of chromothripsis, catastrophic rearrangements in a limited number of chromosomes, vis-a-vis tumor immunity. The collection of manuscripts indicates the complexity of the topic, and further functional studies in the area are warranted. The topic of genomic instability in cancer remains far from entirely explored and understood, despite the advent of new technologies.

## Author contributions

PV and RK are Guest Editors for this Research Topic, they wrote the text, LV and MK contributed with writing, editing and critical reading. All authors contributed to the article and approved the submitted version.
